# Late Pleistocene songbirds of Liang Bua (Flores, Indonesia); the first fossil passerine fauna described from Wallacea

**DOI:** 10.7717/peerj.3676

**Published:** 2017-08-17

**Authors:** Hanneke J.M. Meijer, Rokus Awe Due, Thomas Sutikna, Wahyu Saptomo, Jatmiko, Sri Wasisto, Matthew W. Tocheri, Gerald Mayr

**Affiliations:** 1 University Museum of Bergen, Department of Natural History, University of Bergen, Bergen, Norway; 2 Human Origins Program, Department of Anthropology, National Museum of Natural History, Smithsonian Institution, Washington, DC, United States of America; 3 Pusat Penelitian Arkeologi Nasional, Jakarta, Indonesia; 4 Centre for Archaeological Science, School of Earth and Environmental Sciences, University of Wollongong, Wollongong, New South Wales, Australia; 5 Department of Anthropology, Lakehead University, Thunder Bay, Canada; 6 Ornithological Section, Senckenberg Research Institute, Frankfurt am Main, Germany

**Keywords:** Aves, Passeriformes, Avifauna, Passerines, Wallacea, Southeast Asia

## Abstract

**Background:**

Passerines (Aves: Passeriformes) dominate modern terrestrial bird communities yet their fossil record is limited. Liang Bua is a large cave on the Indonesian island of Flores that preserves Late Pleistocene–Holocene deposits (∼190 ka to present day). Birds are the most diverse faunal group at Liang Bua and are present throughout the stratigraphic sequence.

**Methods:**

We examined avian remains from the Late Pleistocene deposits of Sector XII, a 2 × 2 m area excavated to about 8.5 m depth. Although postcranial passerine remains are typically challenging to identify, we found several humeral characters particularly useful in discriminating between groups, and identified 89 skeletal elements of passerines.

**Results:**

At least eight species from eight families are represented, including the Large-billed Crow (*Corvus* cf. *macrorhynchos*)*,* the Australasian Bushlark (*Mirafra javanica*)*,* a friarbird (*Philemon* sp.), and the Pechora Pipit (*Anthus* cf.* gustavi*)*.*

**Discussion:**

These remains constitute the first sample of fossil passerines described in Wallacea. Two of the taxa no longer occur on Flores today; a large sturnid (cf. *Acridotheres*) and a grassbird (*Megalurus* sp.). Palaeoecologically, the songbird assemblage suggests open grassland and tall forests, which is consistent with conditions inferred from the non-passerine fauna at the site. *Corvus* cf. *macrorhynchos*, found in the *Homo floresiensis*-bearing layers, was likely part of a scavenging guild that fed on carcasses of *Stegodon florensis insularis* alongside vultures (*Trigonoceps* sp.), giant storks (*Leptoptilos robustus*), komodo dragons (*Varanus komodoensis*), and probably *H. floresiensis* as well.

## Introduction

Passerines (Aves: Passeriformes), which include the globally distributed songbirds (Oscines), constitute nearly 60% of extant bird species and occupy almost every terrestrial habitat (Sibley & Monroe, 1990; Jetz et al., 2012). Located within Wallacea, Flores hosts a remarkable avifauna with a number of poorly known endemics. The avifauna includes at least 90 species of passerines (Table 1; Verhoeye & Holmes, 1998; Mees, 2006; Eaton et al., 2016), of which three are endemic to the island; the Flores crow *Corvus florensis,* the Flores Monarch *Symposiachrus sacerdotum,* and the Flores Warbling-flycatcher *Eumyias oscillans*. Ornithological collecting on the island started with the independent arrival of Jan Semmelink from the Rijksmuseum van Natuurlijke Historie (now the Netherlands Biodiversity Center) in Leiden, and Charles Allen, assistant to Alfred Russel Wallace, in 1862. In the years that followed numerous other collectors visited Flores, including Max W.C. Weber, director of the Zoölogisch Museum in Amsterdam and Alfred H. Everett, a collector in service of Lord Rothschild. In the 20th century, the work of Jilis A.J. Verheijen and Erwin Schmutz, two priests who spent decades collecting specimens and making field observations, greatly advanced our ornithological knowledge of Flores (see Mees (2006) for a detailed history of ornithological explorations on Flores). However, despite a century and a half of ornithological attention, basic distributional data for most of the taxa found on Flores is incomplete.

Although a number of skeletal characters distinguishes passerines from non-passerines, their fossil record has received far less attention than that of non-passerines because identifying taxa is challenging due to their small body sizes and perceived skeletal homogeneity. This challenge is compounded further if dealing with isolated and fragmentary postcranial remains. Thus, analyses of avian skeletal remains have focused mostly on non-passerines; however, there are descriptions of passerine osteological characters for certain elements (e.g., Ashley, 1941; Bock, 1962; Jánossy, 1983; Höfling & Alvarenga, 2001; Mayr, 2016) or groups (Tomek & Bocheński, 2000). For Island Southeast Asia, a region with a poor fossil bird record, only twelve passerine taxa (19%) have been identified, in contrast to fifty-one (81%) non-passerine taxa (Meijer, 2014).

**Figure 1:**
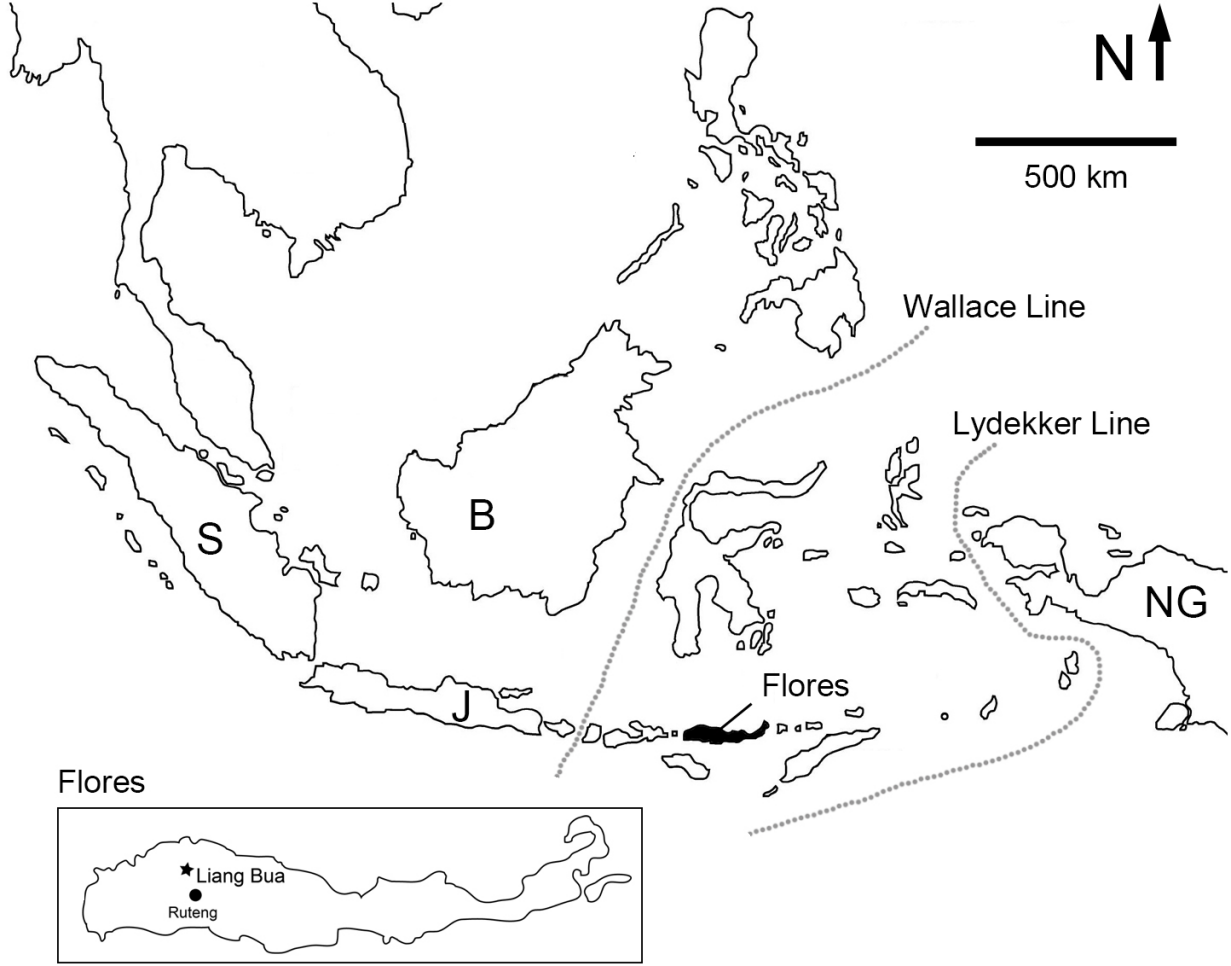
Map of Island Southeast Asia showing the location of Flores. Inset: location of Liang Bua on Flores. Dotted lines denote the western and eastern limits of Wallacea by the Wallace Line and the Lydekker Line, respectively. Abbreviations: B, Borneo; J, Java; NG, New Guinea; S, Sumatra.

Liang Bua is a large, limestone cave on the Indonesian island of Flores (Fig. 1). The site preserves a stratigraphic sequence that encompasses the past 190 thousand years (ka) (Westaway et al., 2007; Sutikna et al., 2016; Morley et al., 2016). Sediments between ∼190 and 50 ka old reveal an impoverished insular fauna that includes small-bodied hominins (*Homo floresiensis* Brown et al., 2004), pygmy proboscideans (*Stegodon florensis insularis* Van den Bergh et al., 2008), komodo dragons (*Varanus komodoensis* Ouwens, 1912), and multiple species of rats, bats and birds, including giant marabou storks (*Leptoptilos robustus* Meijer & Due, 2010) and vultures (*Trigonoceps* sp. Lesson, 1842) (Morwood et al., 2004; Morwood et al., 2005; Van den Bergh et al., 2009; Hocknull et al., 2009; Meijer et al., 2010; Meijer et al., 2013; Meijer et al., 2015; Locatelli, 2011; Veatch, 2015).

**Table 1:** List of extant passerine families and genera from Flores.

Family		Genus	#	Flores endemic
Pittidae	Pittas	*Pitta*	1	
Meliphagidae	Honeyeaters	*Philemon*	1	
		*Lichmera*	2	
Acanthizidae	Australasian warblers	*Gerygone*	1	
Campephagidae	Cuckooshrikes and allies	*Coracina*	2	
		*Lalage*	1	
		*Pericrocotus*	1	
		*Edolisoma*	1	
Pachycephalidae	Whistlers	*Pachycephala*	2	
Oriolidae	Orioles	*Oriolus*	1	
Artamidae	Woodswallows	*Artamus*	1	
Dicruridae	Drongos	*Dicrurus*	1	
Rhipiduridae	Fantails	*Rhipidura*	2^a^	
Laniidae	Shrikes	*Lanius*	2	
Corvidae	Crows and allies	*Corvus*	2	*Corvus florensis*
Monarchidae	Monarchs	*Hypothymis*	1	
		*Terpsiphone*	1	
		*Symposiachrus*	2	*Symposiachrus sacerdotum*
		*Monarcha*	1	
		*Myiagra*	1	
Dicaeidae	Flowerpeckers	*Dicaeum*	4^b^	
Nectariniidae	Sunbirds	*Anthreptes*	1	
		*Cinnyris*	2	
Estrildidae	Finches and Munias	*Amandava*	1	
		*Lonchura*	4	
		*Taeniopygia*	1	
		*Erythrura*	1	
Passeridae	Sparrows	*Passer*	1	
Motacillidae	Pipits and wagtails	*Anthus*	2	
		*Motacilla*	3	
Stenostiridae	Flycatchers	*Culicicapa*	1	
Paridae	Tits	*Parus*	1	
Alaudidae	Larks	*Mirafra*	1	
Cisticolidae	Cisticolas	*Cisticola*	2	
Locustellidae	Warblers	*Locustella*	2	
Acrocephalidae	Reed Warblers	*Acrocephalus*	1	
Timaliidae	Wren-babbler	*Pnoepyga*	1	
Hirundinidae	Swallows and martins	*Petrochelidon*	1	
		*Hirundo*	2	
		*Cecropis*	2	
Phylloscopidae	Leaf warblers	*Seicercus*	6	
Scotocercidae	Scrub warblers	*Tesia*	1	
		*Phyllergates*	1	
		*Horornis*	1	
Zosteropidae	White-eyes and allies	*Heleia*	4	
		*Zosterops*	3	
Sturnidae	Starlings and mynas	*Aplonis*	1	
		*Gracula*	1	
		*Acridotheres*	1^c^	
Muscicapidae	Chats and flycatchers	*Cyornis*	1	
		*Eumyias*	1	*Eumyias oscillans*
		*Brachypteryx*	2	
		*Ficedula*	3	
		*Monticola*	1	
		*Saxicola*	1	
Turdidae	Thrushes	*Zoothera*	1	
		*Geokichla*	3	
		*Turdus*	1	

**Notes.**

# denotes the number of species within each genus.

a Although Verhoeye & Holmes (1998) and Mees (2006) record three species for Flores, the presence of the 3rd (*R. rufifrons*) is not confirmed by Del Hoyo et al. (2017) or Eaton et al. (2016).

b Avibase record five species of flowerpeckers, but the presence of *D. maugei* is not confirmed by Verhoeye & Holmes (1998), Mees (2006), Del Hoyo et al. (2017) or Eaton et al. (2016).

c *A. javanicus* is considered introduced.

Bird remains make up ∼1% of the total number of faunal vertebrate elements at Liang Bua but they are the most species-rich faunal group, with 28 non-passerine species identified thus far (Meijer et al., 2013; Meijer et al., 2015; Sutikna, 2016). Meijer et al. (2013) described a diverse Late Pleistocene non-passerine avifauna that includes swiftlets (*Aerodramus* cf. *fuciphagus* and *Collocalia esculenta*), buttonquails (*Turnix* sp.), plovers (*Pluvialis fulva*), snipes (*Gallinago* sp.), pigeons (*Macropygia* sp., *Ducula* sp.), parrots (*Geoffroyus* cf. *geoffroyus*), owls (*Tyto* sp., *Otus* sp.) and raptors (*Aquila* sp., *Haliastur* cf. *indus*). This non-passerine avifauna reflects a Late Pleistocene environment dominated by wetlands and forest and, to a lesser degree, open woodland. Avian extinctions on Flores during the Late Pleistocene appear limited to *L. robustus* and *Trigonoceps* sp., but the disappearance of these taxa did not significantly alter avian community structure overall (Meijer et al., 2015). In order to better understand the avifauna and palaeoecology of Liang Bua, as well as the biogeography of Wallacean bird communities, we aimed to identify passerine remains from the Late Pleistocene sediments at Liang Bua, which would constitute the first fossil assemblage of songbirds described from Wallacea.

## Materials and Methods

Sector XII is one of twenty-eight areas that have been excavated at Liang Bua. It is a 2 × 2 m square located in the middle rear of the cave that was excavated to 8.45 m depth from the present day cave floor in 2007 (Permit numbers PW.007/885/P3AN/DKP/II/07 (March 15, 2007) and PW.007/2574/P3AN/DKP/VI/07 (June 5, 2007)). Excavations were authorized by the Pusat Penelitian Arkeologi Nasional (Jakarta, Indonesia). The first excavated unit (Spit 1) was 15 cm deep with subsequent spits proceeding in 10 cm-deep intervals while following stratigraphic layers. Between 2 and 8.45 m depth, Sector XII preserves undisturbed Late Pleistocene sediments that are ∼190–50 ka old (Westaway et al., 2007; Sutikna et al., 2016). These sediments are capped by a volcaniclastic mass flow deposit (Tephra 3, or T3), a key stratigraphic layer in the Liang Bua depositional sequence, that varies in thickness (∼0.5–0.75 m) throughout the entire sector (Sutikna et al., 2016).

Excavation of Sector XII recovered 235 bird bones from sediments beneath T3 using dry and wet-sieving through 2 mm mesh. Of these, 89 elements were identified as passerines (for non-passerine elements see Meijer et al., 2015). Further taxonomic assignment of these remains was conducted using extant comparative material from the Senckenberg Research Institute (Frankfurt, Germany), Bergen University Museum (Bergen, Norway), and the Smithsonian Institution’s National Museum of Natural History (Washington DC, USA) (see Table S1). Unfortunately, available comparative material for this study was limited: no modern skeletal specimens from Flores were available for this study, and many of the taxa that occur on Flores are poorly represented in avian skeleton collections (out of the 90 species of passerines present on Flores, specimens of only 35 species were available for comparisons). The Smithsonian Institution’s National Museum of Natural History does hold good samples of birds from other Indonesian islands, such as Sulawesi, Java and the Moluccas, and in many cases, specimens from species within the same genus were used. However, this restricted the level to which we were able to identify fossil specimens. Undoubtedly, as more comparative material from Flores becomes available, the number of fossil specimens and species identified will rise accordingly. The list of passerine taxa reported here should therefore be regarded as an initial exploration of Quaternary passerines in Southeast Asia. The systematic framework mostly followed Dickinson & Christidis (2014), with the exception of Locustellidae, for which we followed Gill & Donsker (2017). Anatomical nomenclature follows Baumel & Witmer (1993). Specimens were catalogued as ‘LB-Av-##’, where ‘LB’ refers to Liang Bua, ‘Av’ to Aves, and ‘##’ is a unique number. All specimens are curated at the National Research Center for Archaeology (Pusat Penelitian Arkeologi Nasional) in Jakarta, Indonesia.

## Results

### Passerine humeral morphology

Proximal humeral morphology, particularly the presence/absence and morphology of the fossae pneumotricipitales dorsalis et ventralis varies markedly among passerines (Ashley, 1941; Bock, 1962). The morphology of the pneumatic fossae in passerines varies from the single-fossa condition, in which the pneumatic fossa (here referred to as the fossa pneumotricipitalis dorsalis) lies directly distal to the ventral tubercle and posterior to the medial bar, to the double fossa condition, in which a second fossa (the fossa pneumotricipitalis ventralis) develops under the head of the humerus and ultimately merges with the fossa pneumotricipitalis dorsalis (Ashley, 1941; Bock, 1962). Although the single fossa condition appears to be the primitive condition and the development of the fossa pneumotricipitalis ventralis an advanced trait, Bock (1962) argues that the presence of the double fossa condition not necessarily translates in a species being advanced.

**Figure 2:**
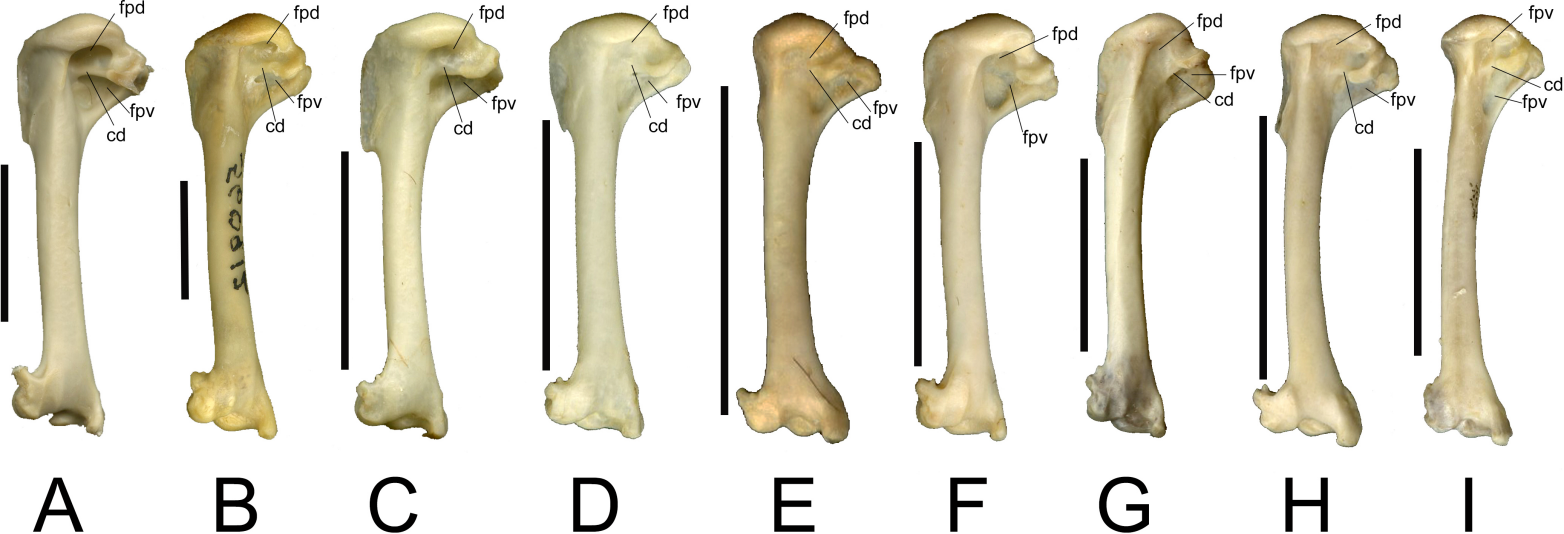
Left humeri (all in caudal view) of selected extant passerine taxa examined in this study. All humeri are scaled to approximately the same length to illustrate proportional shape differences, scale bars indicate 1 cm. (A) *Turdus obscurus* (Turdidae); (B) *Acridotheres cristatellus* (Sturnidae); (C) *Pachycephala pectoralis* (Pachycephalidae); (D) *Rhipidura javanica* (Rhipiduridae); (E) *Zosterops montana* (Zosteropidae); (F) *Anthus gustavi* (Motacillidae); (G) *Mirafra javanica* (Alaudidae); (H) *Brachypteryx montana* (Muscicapidae); (I) *Megalurus timoriensis* (Locustellidae). Abbreviations; cd, crus dorsale fossae; fpd, fossa pneumotricipitalis dorsalis; fpv, fossa pneumotricipitalis ventralis.

In our sample of Southeast Asian passerines, we found the condition of the fossae useful in distinguishing between families (see Fig. 2). Care was taken to include multiple species per family, as morphological variation may also be present within families. Turdidae (Fig. 2A) and Sturnidae (Fig. 2B), the species of both of which are relatively large for Southeast Asian passerines, typically display the double fossa condition, with a very deep fossa pneumotricipitalis dorsalis that is separated from a deep fossa pneumotricipitalis ventralis by a distinct crus dorsale fossae. Jánossy (1983) noted that in European Sturnidae, the medial bar (i.e., the crus dorsale fossae) separates the two fossae, whereas in Turdidae, the two fossae are confluent with a reduced medial bar. A survey of several Asian species of *Turdus* (*T. ruficollis, T. poliocephalus, T. chrysolaus, T. naumanni, T. obscurus, T. merula mandarinus*) indicates that in general, the medial bar is more reduced in *Turdus* species than in similar sized sturnids, but there is variation in the degree of reduction of the medial bar: in *T. obscurus,* the medial bar is slightly more pronounced than in other *Turdus* species. In contrast to *Turdus,* a well-developed crus dorsale fossae was also observed in *Zoothera dauma*. Within Sturnidae, the taxa *Sturnus, Acridotheres*, *Rhabdornis* and *Leucopsar* all display a large and deep fossa pneumotricipitalis dorsalis that is separated from the fossa pneumotricipitalis ventralis by a well-developed crus dorsale fossae. In the sturnids *Aplonis*, *Mino*, *Ampeliceps*, *Streptocitta* and *Gracula*, the dorsal fossa is much shallower and narrower. *Basilornis* attains an intermediate position; the fossa pneumotricipitalis dorsalis is deep but narrower than in *Acridotheres*. The fossa pneumotricipitalis dorsalis is also distinct in Pachycephalidae (Fig. 2C), Rhipiduridae (Fig. 2D) and Zosteropidae (Fig. 2E), but less deep than in Turdidae and Sturnidae. In Motacillidae (Fig. 2F), the fossa pneumotricipitalis dorsalis merges with the fossa pneumotricipitalis ventralis forming one large fossa. The crus dorsale fossae is reduced and limited to the ventral side of the crista bicipitalis. Alaudidae (Fig. 2G) display a shallow fossa pneumotricipitalis dorsalis and the proximal humerus is relatively elongated.

In addition to the condition of the fossae, Jánossy (1983) mentions the presence or absence of pneumatic foramina in the fossa pneumotricipitalis ventralis (the fossa pneumoanconaea in Jánossy, 1983) as a key feature in distinguishing passerine families. In general, our observations agree with his work. For instance, pneumatic foramina are generally present in Alaudidae, and generally absent in Sturnidae and Turdidae. However, the presence of pneumatic foramina in *Acridotheres javanicus,* and their absence in specimens of *Motacilla flava* studied here indicate that this character displays variation within families and even within species. Its value for identifying isolated passerine postcranial remains to species level should therefore be considered with caution.

*Brachypteryx* (Muscicapidae) (Fig. 2H) and *Megalurus* (Locustellidae) (Fig. 2I) display the single fossa condition, with a fossa pneumotricipitalis dorsalis that is broad yet shallow. In both taxa, the humerus is slender with a curved shaft and a distinctly shortened proximal end, and this is especially pronounced in *Megalurus*. This particular humeral morphology, including a wide incisura capitis and a head that is slanted towards the deltoid crest, was also described by Feduccia & Olson (1982) for Rhinocryptidae and by Rich, McEvey & Baird (1985) for the lyrebirds (*Menura*) and scrubbirds (*Atrichornis*), and attributed to a convergence to a terrestrial lifestyle. Commonly named shortwings, *Brachypteryx* features shortened wings, inhabits the forest floor and understory, and hops between branches (Coates & Bishop, 1997). The shortened proximal end is most extreme in *Megalurus*, where the humerus is slender and curved. Many species of *Megalurus* fly reluctantly or weakly and only short distances when flushed (Coates & Bishop, 1997; Del Hoyo et al., 2017). Whereas *Brachypteryx* feeds both on the ground and in the lower branches, necessitating a minimal amount of flying, the extreme morphology observed in *Megalurus* reflects its terrestrial preferences. As for the Rhinocryptidae, lyrebirds and scrubbirds, the similarities in morphology between these two distantly related species are due to convergence owing to their terrestrial habits.

### Species account

At least eight passerine species—six of which are extant taxa known from Flores—from eight different families (Table 2) were recorded from postcranial skeletal elements, extending the total number of avian species in these deposits at Liang Bua to thirty-three.

**Table:** 

**Figure 3:**
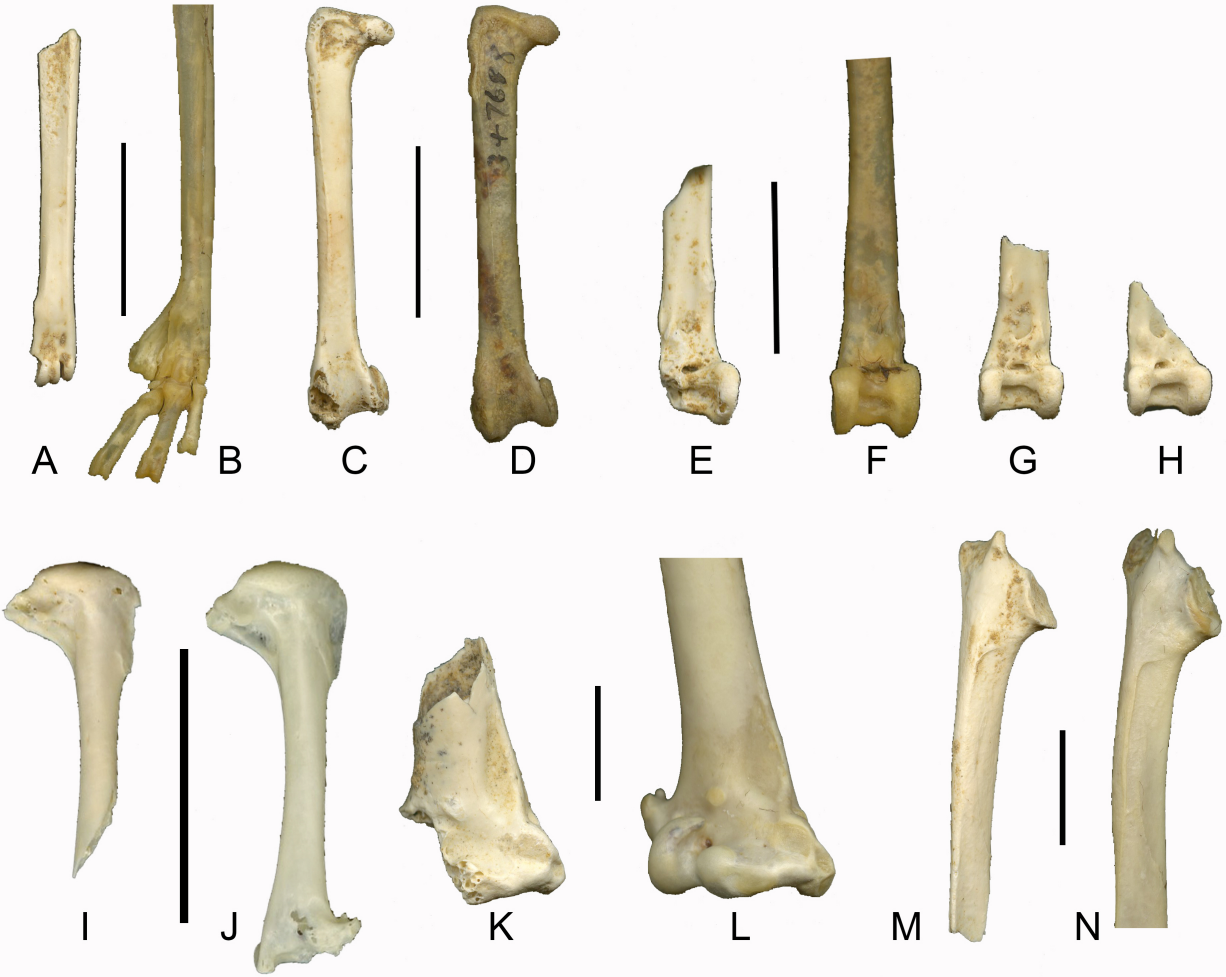
Late Pleistocene passerines from Liang Bua (scale bars 1 cm). (A) Left tarsometatarsus of *Philemon* sp. (LB-Av-740), (B) left tarsometatarsus of *P. buceroides* (NMNH 347688); (C) right femur of *Philemon* sp.(LB-Av-795), (D) right femur of *P. buceroides* (NMNH 347688); (E) right tibiotarsus of cf. *Philemon* (LB-Av-857), (F) right tibiotarsus of *P. buceroides* (NMNH 347688), (G) left tibiotarsus (LB-Av-726) of cf. *Philemon*, (H) right tibiotarsus (LB-Av-775) cf. *Philemon*; (I) right humerus of *Rhipidura* sp. (LB-Av-762), (J) right humerus of *R. albicollis* (NMNH 620568); (K) distal fragment of right humerus of *Corvus* cf. *macrorhynchos* (LB-Av-856), (L) right humerus of *C. macrorhynchos* (NMNH 641775); (M) right scapula of *Corvus* cf. *macrorhynchos* (LB-Av-766), (N) right scapula of *C. macrorhynchos* (NMNH 641775).

Aves Linnaeus, 1758
Passeriformes (Linnaeus, 1758)
Meliphagidae Vigors, 1825
*Philemon*Vieillot, 1816
*Philemon* sp.
(Fig. 3A)

**Material**: A distal left tarsometatarsus lacking the trochlea metatarsi II (LB-Av-740).

**Table 2:** Passerines identified from Late Pleistocene deposits at Liang Bua’s Sector XII.

Family	Taxon		NISP	Status	Habitat affinities	Dietary preferences
Meliphagidae	*Philemon* sp.	Friarbird	1	Resident	Wooded habitats, flowering trees	Mainly nectivorous
	cf. *Philemon*		4			
Rhipiduridae	*Rhipidura* sp.	Fantail	1	Resident?	Forest habitats	Insects
Corvidae	*Corvus* cf. *macrorhynchos*	Large-billed Crow	3	Resident	Favours forest edge and clearings near rivers and in coastal lowlands	Omnivorous (including carrion), known to feed with vultures on carcasses
Motacillidae	*Anthus* cf. *gustavi*	Pechora Pipit	2	Migratory	Winter visitor (Sep–March) to Wallacea, found in wet grassy areas and open woodland. Forages on the ground	Mainly insects
Alaudidae	*Mirafra javanica*	Australasian Bushlark	1	Resident	Open habitats, such as grasslands with scattered bushes and trees. Lowlands and middle elevations. Feeds and nests on the ground	Seeds and insects
Locustellidae	*Megalurus* sp.^a^	Grassbird	2	Absent from Flores; Resident on Sumba and Timor	Open habitats, such as reedbeds and grasslands with scattered shrubs, particularly in riverine floodplains	Insects
Sturnidae	cf. *Acridotheres*^b^	Myna	1	?	Range of wooded habitats	Diet includes wide range of invertebrates, fruits, and seeds
Turdidae	*Turdus* cf. *obscurus*	Eye-browed Thrush	1	Migratory	Winter visitor, open forest, secondary forest, and hillside scrub	Fruits and insects
	Turdidae indet.		2			
	TOTAL		18			

**Notes.**

NISP number of identified specimens

a This genus is currently not known from Flores.

b *Acridotheres* is considered recently introduced.

**Horizon**: Late Pleistocene (spit 29).

**Geological Age:** ∼60–50 ka.

**Measurements**: Preserved length, 25.5; minimum width of shaft, 2.17; preserved distal width, 3.10.

**Remarks**: This bone has a distinct crista plantaris lateralis and the trochlea metatarsi IV is in line with the shaft (i.e., it does not flare laterally). The fossa metatarsi I and the foramen vasculare distale are placed relatively more distal than in similar-sized passerines. It most resembles the tarsometatarsus of honeyeaters (Meliphagidae), displaying five of the seven key honeyeater characteristics (Boles, 2005) (two of the seven characteristics pertain to features in the proximal end which is not preserved in the specimen): (1) the distal end, particularly the section bearing the trochleae metatarsorum, is compressed and elongated; (2) in lateral/medial view, the distal end of the bone displays a plantar bend; (3) the medial rather than the lateral rim of the trochlea metatarsi III projects furthest distally; (4) the lateral rim of the trochlea metatarsi III reaches further distally than the trochlea metatarsi IV; and (5) caudally, the fossa metatarsi I is large, deep, and occupies the medial half of the shaft surface. LB-Av-740 also displays relatively long trochleae (longer than those figured in Boles, 2005) and its trochlea metatarsi IV is shorter than the lateral rim of the trochlea metatarsi III, suggesting that, within honeyeaters, it is most similar to *Philemon* spp. In size, it approximates *P. buceroides* (minimum width of the shaft 2.24 mm) and is larger than the other genus of honeyeaters on Flores, *Lichmera.*

**Table:** 

Meliphagidae
cf. *Philemon*
(Figs. 3C, 3E, 3G and 3H)

**Material**: Two distal right tibiotarsi (LB-Av-775 and LB-Av-857), one distal left tibiotarsus (LB-Av-726), and a right femur (LB-Av-795).

**Horizon**: Late Pleistocene (spits 38, 34, 33 and 28).

**Geological Age:** ∼120–60 ka (spits 38–33) and 60–50 ka (spit 28).

**Measurements**: LB-Av-775, preserved length, 8.28, distal width, 4.85; LB-Av-857, preserved length 18.12, preserved distal width, 4.53; LB-Av-726, preserved length, 10.82, distal width, 4.88; LB-Av-795, length, 29.6; proximal width, 6.10; distal width, 5.63.

**Remarks**: The tibiotarsus shafts and distal ends are wide, indicating a fairly large species. The condyli are parallel to each other but the condylus medialis is slightly narrower than the condylus lateralis. The lateral and medial border of the condylus lateralis and medialis, respectively, project beyond the margins of the shaft. On the proximal border of the condylus lateralis, a short, proximodistally oriented ridge connects the condylus lateralis to the pons supratendineus, which is higher than wide. The sulcus extensorius is located centrally on the shaft. A small foramen is situated medial to the distal opening of the canalis extensorius, which is situated just below the level of the proximal border of the condylirather than more proximally as in many passerines. Cranially, the incisura intercondylaris is relatively wide (e.g., wider than in *Turdus*, *Sturnus*, *Alauda*, *Coracina* and *Corvus*) and has a wide and deep rectangular attachment for the ligamentum tibiometatarsale intercondylare (but not as deep as in woodpeckers). The proximal border of the incisura intercondylaris forms a distinct ridge, and the distal opening of the canalis extensorius lies somewhat sunken behind it. Medially, the ridge connects with the condylus medialis’ proximal border and is separated from the shaft by a depression. Overall, these tibiotarsi differ from those of most passerines, but are most similar to those of *Philemon* spp. in the wide shaft and incisura intercondylaris, and the distinct proximal ridge. Although the width of the three distal tibiotarsi varies, their size range is smaller than in the single specimen of *P. buceroides* (distal width 5.31 mm) that was available for comparison. However, the distal articular ends of the Liang Bua tibiotarsi are more compressed proximodistally than in *Philemon,* so we refer these specimens to cf. *Philemon* rather than *Philemon* sp. The femur is *Turdus-* sized, but more robust. Its proximal end is flattened and the crista trochanteris does not project proximally, similar to *Philemon* and unlike in, for instance, Laniidae. Cranially, the crista trochanteris is thick and continues on the shaft further distally than in *Turdus.* Distally, the medial condyle is high proximodistally, similar to *Philemon* and unlike *Turdus*, and the impressio ansae m. iliofibularis is small and placed laterally, similar to *Philemon*, whereas in *Turdus* it is facing caudally and is large and round. The specimen agrees in size and morphology with *Philemon,* but is shorter than *P. buceroides* (30.5 mm).

**Table:** 

Rhipiduridae Sundevall, 1872
*Rhipidura*Vigors & Horsfield, 1827
*Rhipidura* sp.
(Fig. 3I)

**Material**: A right humerus lacking the distal end (LB-Av-762).

**Horizon**: Late Pleistocene (spit 31).

**Geological Age:** ∼60 ka.

**Measurements**: Preserved length, 11.82; proximal width, 4.96.

**Remarks**: LB-Av-762 displays a deep and pneumatic fossa pneumotricipitalis ventralis and a shallow fossa pneumotricipitalis dorsalis. Overall, the proximal end is proximodistally shortened, the crista deltopectoralis extends further down the shaft than the bicipital shelf, and the caput humeri is low. Together, these features distinguish this humerus from that of many Southeast Asian passerine families, including Turdidae, Sturnidae, Estrildidae, Passeridae, Motacillidae, Dicaeidae, Cisticolidae, Muscicapidae and Zosteropidae. There is a slight ‘dent’ at the distal end of the fossa pneumotricipitalis ventralis where it borders the shaft, which is also seen in Pachycephalidae, but LB-Av-762 differs in having a lower caput humeri (more rounded in Pachycephalidae) and a proportionally shorter crista deltopectoralis. Compared with Alaudidae, which also display a shallow fossa pneumotricipitalis dorsalis and pneumatic foramina in the fossa pneumotricipitalis ventralis, the caput humeri is lower and less rounded while the crista deltopectoralisand bicipital shelf do not extend as far distally along the shaft. Compared with Laniidae, the distal edge of the caput humeri is more horizontal in Laniidae whereas it is slanted mediodistally in LB-Av-762.

Overall, this humerus agrees best with that of Rhipiduridae, specifically *Rhipidura,* which has smaller humeri than does *Terpsiphone*, and we therefore refer this specimen to *Rhipidura* sp. No specimens of *R. diluta* or *R. dryas,* the two species of fantail on Flores (Eaton et al., 2016), were available for comparisons. In size, this specimen is smaller than *R. albicollis* (humeral length 15.6–17.06 mm; proximal width 5.2–5.48) and *R. javanica* (humeral length 17.0 mm; proximal width 5.8), both of which occur in the region, but is somewhat larger than *R. rufifrons* (humeral length 12.99–13.01 mm; proximal width 4.23–4.31). The Rufous Fantail *R. rufifrons* was reported from Flores by Mees (2006) and Verhoeye & Holmes (1998), but is now often considered conspecific with the Arafura Fantail *R. dryas* (Del Hoyo et al., 2017; Eaton et al., 2016).

**Table:** 

**Figure 4:**
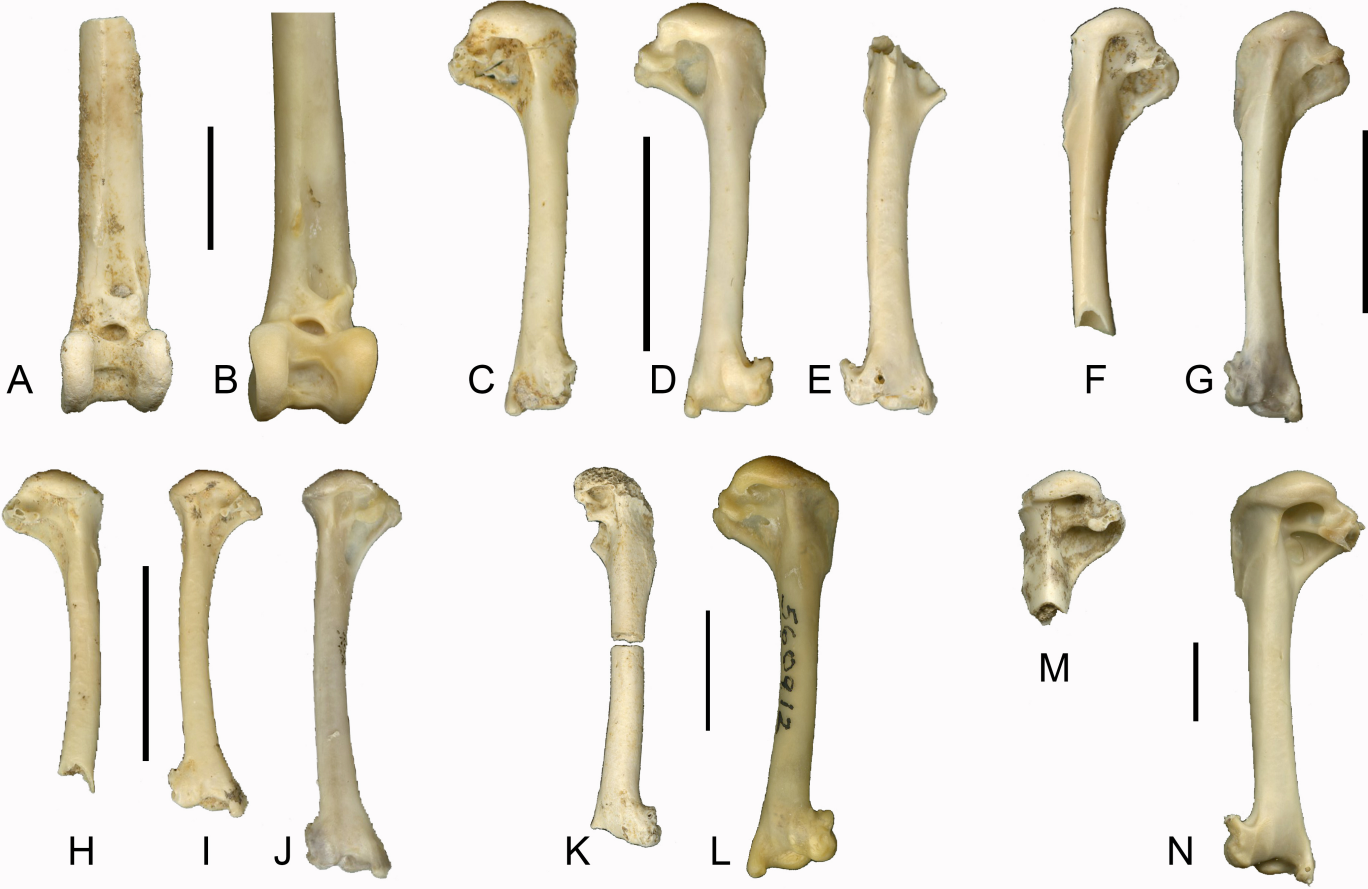
Late Pleistocene passerines from Liang Bua, continued (scale bars 1 cm). (A) left tibiotarsus of *Corvus* cf. *macrorhynchos* (LB-Av-835); (B) left tibiotarsus of *C. macrorhynchos* (NMNH 641775); (C) right humerus of *Anthus* cf. *gustavi* (LB-Av-836); (D) right humerus of *A. gustavi* (NMNH 613109); (E) left humerus of *A*. *gustavi* (LB-Av-1306); (F) left humerus of *Mirafra javanica* (LB-Av-908); (G) left humerus of *M. javanica* (NMNH 612719); (H) right humerus of *Megalurus* sp. (LB-Av-799); (I) left humerus of *Megalurus* sp. (LB-Av-779); (J) left humerus of *M. timoriensis* (NMNH 561990); (K) right humerus of cf. *Acridotheres* (LB-Av-930); (L) right humerus of *A. c. cristatellus* (NMNH 560912); (M) left proximal humerus of *Turdus* cf. *obscurus* (LB-Av-907); (N), left humerus of *T. obscurus* (NMNH 611772).

Corvidae Leach, 1820
*Corvus*Linnaeus, 1758
*Corvus* cf. *macrorhynchos*Wagler, 1827
(Figs. 3K, 3M and 4A)

**Material**: A right scapula (LB-Av-766), a distal right humerus (LB-Av-856), and a distal left tibiotarsus (LB-Av-835).

**Horizon**: Late Pleistocene (spits 38, 37, 32).

**Geological Age:** ∼120–60 ka.

**Measurements**: LB-Av-835, preserved length, 56.5; distal width, 9.1; distal depth, 8.4. LB-Av-766, preserved length, 35.9; distance from facies articularis humeralis to tuberculum coracoideum, 11.4; distance from facies articularis humeralis to acromion, 9.7; LB-Av-856, preserved length 23.36; preserved distal width 11.38.

**Remarks**: These remains are from a very large passerine. On the distal humerus, the fossa brachialis is shallow. The condylus dorsalis is broken off, but the onset of the processus supracondylaris dorsalis is visible. The processus flexorius projects not as far distally as in other passerine groups, and in combination with its large size, the specimen is assigned to Corvidae. The fragmentary nature of the humerus prevents any meaningful measurements to be taken, but it appears smaller than that of *C. corax,* larger than that of *C. enca,* and *C. typicus,* and similar in size to that of *C. macrorhynchos, C. orru* and *C. validus*.

The scapula lacks the distal third and the tip of the medial process of the acromion is broken off. The lateral acromion process is pronounced and pointed, extending slightly beyond the broken tip of the medial process, suggesting that the two were likely equal in length, which is characteristic of *Corvus* (Tomek & Bocheński, 2000). In many other passerine groups, such as Turdidae, Sturnidae, Lanidae, Motacillidae and Oriolidae, the lateral acromion process projects beyond the medial process. The width across the articular surface, from the medial process of the acromion to the facies articularis humeralis, measures 11.5 mm, in which it is larger than *C. typicus* (9.1 mm) and *C. enca* (9.6 and10.1 mm) and similar in size to *C. macrorhynchos* (10.5, 11.5 and 11.9 mm) and *C. validus* (11.7 and 13 mm). The specimen is similar in size to *C. orru,* but in this species, the facies articularis humeralis and lateral acromion process are set more apart than in the fossil specimen. No specimens of *C. florensis*, endemic to Flores (Verhoeye & Holmes, 1998), were available for comparison but this species is quite small (Madge, 2017c).

The tibiotarsus preserves the distal end and part of the shaft. The shaft only minimally flares in width as it meets the condyles. The lateral and medial ridges of the tuberculum retinaculi fibularis are separated from the condylus lateralis by a notch. The tuberositas retinaculi extensoris is located distally to the tuberositas retinaculi fibularis and these do not overlap in position. The condyli lateralis et medialis are parallel to each other and equal in height. The lateral condyle is slightly wider than the medial one. The incisura intercondylaris is wide with a deep pit. The distal articulation of LB-Av-835 is wider than that of *C. enca* (7.6 and 8.1 mm), smaller than that of *C. orru* (9.7 and 9.2 mm) and *C. validus* (9.4 and 10.3 mm), and overlaps with *C. macrorhynchos* (8.4, 9.2 and 9.3 mm). No tibiotarsus of *C. typicus* was available for comparison.

The humerus and scapula overlap in size with *C. macrorhynchos, C. orru* and *C. validus*, whereas the tibiotarsus is smaller than *C. orru* and *C. validus.* Assuming that the specimens represent a single species, and given that *C. macrorhynchos* occurs on Flores (Coates & Bishop, 1997) and *C. orru* or *C. validus* currently do not (both are known from the Lesser Sundas but not from Flores), we tentatively refer these specimens to *C.* cf. *macrorhynchos*.

**Table:** 

Motacillidae Horsfield, 1821
*Anthus*Bechstein, 1805
*Anthus* cf. *gustavi*Swinhoe, 1863
(Figs. 4C and 4E)

**Material**: A right humerus (LB-Av-836), and a left humerus (LB-Av-1306) missing the proximal end.

**Horizon**: Late Pleistocene (spits 43 and 36).

**Geological Age:** ∼120–60 ka.

**Measurements**: LB-Av-836, length, 19.39; proximal width, 5.82; preserved distal width, 4.04; LB-Av-1306, preserved length 17.35; distal width, 4.24.

**Remarks**: LB-Av-836 displays a very deep fossa pneumotricipitalis dorsalis, which lacks pneumatic foramina, that is confluent with the fossa pneumotricipitalis ventralis. The crus dorsale fossae is strongly reduced and nearly absent, and the floor of the fossa pneumotricipitalis (i.e., the bicipital shelf) is very thin. Although humerus LB-Av-1306 lacks the most proximal end, the remaining part of the crista bicipitalis is very thin and resembles LB-Av-836 in that regard. Distally, the fossa for the m. brachialis is deep and the processus supracondylaris dorsalis relatively long. A confluence of the fossae pneumotricipitalis dorsalis and ventralis is present in only a few passerine groups, including Prunellidae, Aegithalidae, Remizidae, and Motacillidae (Bock, 1962; Jánossy, 1983). The crus dorsale fossae is nearly absent in LB-Av-836 (a distinguishing characteristic of Motacillidae), in both specimens the floor of the fossa pneumotricipitalis (i.e., the bicipital shelf) is very thin, the fossa for the m. brachialis is deep, and in LB-Av-1306 the processus supracondylaris dorsalis relatively long. Further comparisons revealed that a very thin, almost translucent bicipital shelf is also characteristic of Motacillidae. Only two genera of Motacillidae are present on Flores, *Motacilla* and *Anthus,* with 2 and 3 species respectively. Both genera are rather similar in morphology (also see Jánossy, 1983), but we found *Anthus* to have a slenderer shaft and relatively narrower proximal end. In this respect, the fossil specimens agree better with *Anthus* than *Motacilla.* Jánossy (1983) described a tendency for pneumatisation of the fossa pneumotricipitalis dorsalis in certain species, including *M. flava*, but we did not observe pneumatic foramina in either *Anthus* or *Motacilla.* Both fossil specimens are larger than *Motacilla flava* (16.6–18.3 mm in length) and *M. cinerea* (17.15 and 17.8 mm), but are similar in size and morphology to *Anthus gustavi* (19.15–19.3 mm in length). No specimens of *A. novaeseelandiae* were available for study, but since this species is larger than *A. gustavi*, we tentatively assign LB-Av-836 and −1306 to the latter.

**Table:** 

Alaudidae Vigors, 1825
*Mirafra*Horsfield, 1821
*Mirafra javanica*Horsfield, 1821
(Fig. 4F)

**Material**: A proximal left humerus (LB-Av-908).

**Horizon**: Late Pleistocene, (spit 42).

**Geological Age:** ∼120–60 ka (spit 42).

**Measurements**: Preserved length, 18.06; preserved proximal width, 6.14.

**Remarks**: This bone is from a medium-sized passerine. The proximal end is rather narrow and proximodistally elongated. The caput humeri is rounded and relatively high. The fossa pneumotricipitalis dorsalis is distinct and relatively narrow and shallow. In this respect, it differs from families that have a deeper fossa pneumotricipitalis dorsalis (such as Turdidae, Sturnidae, Estrildidae, Passeridae and Zosteropidae) and those that have a shallower fossa (Oriolidae, Laniidae and Corvidae). In Rhipiduridae and Monarchidae, the fossa pneumotricipitalis dorsalis is also shallow but the crista deltopectoralis is relatively shorter. The fossa pneumotricipitalis ventralis is pneumatic, which in combination with the relatively shallow fossa pneumotricipitalis dorsalis, is typical for Alaudidae (i.e., larks) (Jánossy, 1983). Only two species of lark currently occur in the region: the Australasian bushlark *Mirafra javanica* and the Eurasian Skylark *Alauda arvensis* (Eaton et al., 2016). Comparisons to extant material of larks were limited to these two species. Morphological differences between *Alauda arvensis* and *Mirafra javanica* are slight, with *A. arvensis* being larger and overall more robust and having a slightly wider (medio-laterally) bicipital shelf, whereas in *M. javanica* the caput humeri is marginally more rounded. In these aspects, the fossil specimen agrees best with *M. javanica.*

**Table:** 

Locustellidae Bonaparte, 1854
*Megalurus*Horsfield, 1821
*Megalurus* sp.
(Figs. 4H and 4I)

**Material**: A left humerus (LB-Av-779) and a right humerus lacking the distal end (LB-Av-799).

**Horizon**: Late Pleistocene (spits 34 and 33).

**Geological Age:** ∼120–60 ka.

**Measurements**: LB-Av-779, length, 17.97; proximal width, 4.79; distal width, 3.73; LB-Av-799, preserved length 16.61; proximal width, 5.07.

**Remarks**: Both bones display a distinct morphology with a slender and gently curved shaft and a shortened proximal end. The fossa pneumotricipitalis ventralis is deep and does not contain pneumatic foramina in LB-Av-779, but contains one small pneumatic foramen in LB-Av-799. The crista deltopectoralis is short, the incisura capitis is wide, and the fossa pneumotricipitalis dorsalis distinctly excavated but not as deep as in Turdidae and Sturnidae. These features distinguish the specimens from many passerine families, but do resemble the humeral morphology of lyrebirds (*Menura*), scrubbirds (*Atrichornis*) and the South American Rhinocryptidae (Feduccia & Olson, 1982; Rich, McEvey & Baird, 1985), and a comparable morphology of the humerus was also found within Locustellidae. This family of Old World warblers includes a number of species that are mostly terrestrial and reluctant to fly (Del Hoyo et al., 2017), which probably accounts for their distinctive humeral morphology. Within Locustellidae, *Bradypterus* possesses a more reduced crus dorsale fossae, and, on the distal articulation, a processus supracondylaris dorsalis that is set relatively more distally on the shaft. The specimen resembles *Locustella*, but differs in having a deeper fossa pneumotricipitalis dorsalis and a more undercut caput humeri. In all of these features, the specimens are most similar to *Megalurus*, particularly *M. palustris*, although LB-Av-779 is smaller in size than that species and *M. timoriensis*. Specimen LB-Av-799 is slightly larger. Because there is significant size variation between and within species of *Megalurus* and both specimens fall within that observed in *Megalurus*, we refer both to *Megalurus* sp.

**Table:** 

Sturnidae Rafinesque, 1815
*Acridotheres*Vieillot, 1816
cf. *Acridotheres*
(Fig. 4K)

**Material**: A right humerus in two pieces, with the lateral half of the bicipital shelf, just lateral of the crus dorsale fossae, as well as the condyles and the processus supracondylaris dorsalis missing (LB-Av-930).

**Horizon**: Late Pleistocene (spit 45).

**Geological Age:** ∼120–60 ka.

**Measurements**: LB-Av-930, preserved length 31.0; preserved proximal width, 5.72; preserved distal width, 5.78.

**Remarks**: This humerus is from a fairly large passerine. The deep fossa pneumotricipitalis dorsalis is separated from the fossa pneumotricipitalis ventralis by a well-developed crus dorsale fossae. This distinguishes the bone from other groups with larger-sized species, such as Corvidae, Meliphagidae, Dicruridae and Campephagidae, and agrees with Sturnidae and Turdidae. The ventral portion of the crista bicipitalis is broken off, showing the deeply excavated fossa pneumotricipitalis ventralis that continues into the shaft of the bone but does not contain any pneumatic foramina. The crus dorsale fossa is more solid and developed, and the two fossae pneumotricipitales more separated than in most Turdidae, except for *Zoothera dauma.* LB-Av-930 is larger than *Sturnus*, *Rhabdornis* and *Leucopsar,* and agrees in size and morphology with both *Acridotheres* spp. and *Zoothera dauma,* neither of which are part of the native Flores avifauna. *Acridotheres javanicus* is recently introduced (Verhoeye & Holmes, 1998) whereas *Z. dauma* does not occur on Flores*.* Both taxa are rather similar morphologically but are distinguishable by a deeper fossa pneumotricipitalis dorsalis, a slightly shorter crista deltopectoralis and a less robust caput humerus (in medial view) in *Acridotheres*. Moreover, *Acridotheres* appears to have a very deep fossa pneumotricipitalis ventralis. In *A. javanicus,* pneumatic foramina are present in this fossa, but we fail to find any in other species of *Acridotheres.* Therefore we tentatively assign LB-Av-930 to cf. *Acridotheres.*

**Table:** 

Turdidae Rafinesque, 1815
*Turdus*Linnaeus, 1758
*Turdus* cf. *obscurus*Gmelin, 1789
(Fig. 4M)

**Material**: A proximal left humerus (LB-Av-907).

**Horizon**: Late Pleistocene (spit 42).

**Geological Age:** ∼120–60 ka.

**Measurements**: Preserved length, 9.96; proximal width, 6.80.

**Remarks**: The fossae pneumotricipitalis dorsalis and ventralis are very deep but do not contain pneumatic foramina, similar to the condition observed in Turdidae and Sturnidae (Jánossy, 1983). The bone is assigned to Turdidae rather than Sturnidae based on the crus dorsale fossae, which is reduced in comparison to Sturnidae, and the two fossae pneumotricipitalis that are distally confluent, similar to many (smaller) *Turdus* species. The specimen differs from similarly-sized species of *Zoothera* in that the crista deltopectoralis is somewhat longer, and in that the fossa pneumotricipitalis dorsalis is narrower. It is therefore referred to *Turdus.* The specimen has a slightly more pronounced medial bar, which appears less reduced than in other *Turdus* species. In this respect it corresponds most to *T. obscurus*, but to acknowledge the variation of this feature within *Turdus,* we refer this specimen to *T.* cf. *obscurus.*

Turdidae indet.

**Material**: A right proximal humerus (LB-Av-727) and a left proximal humerus (LB-Av-804).

**Horizon**: Late Pleistocene (spits 29, 34).

**Geological Age:** ∼120–60 ka (spit 34) and 60–50 ka (spit 29).

**Measurements**: LB-Av-727, preserved length, 9.87; proximal width, 8.88. LB-Av-804, preserved length, 18.44; proximal width, 8.62.

**Remarks**: These two humeri display a very deep fossa pneumotricipitalis dorsalis and ventralis, both of which are not pneumatic. In this, they resemble Sturnidae and Turdidae, but the reduced crus dorsale fossae in both of them agrees more with Turdidae. In size, these specimens are larger than that of most *Zoothera* species and in the size range of *T. obscurus*, but are more robust.

**Figure 5:**
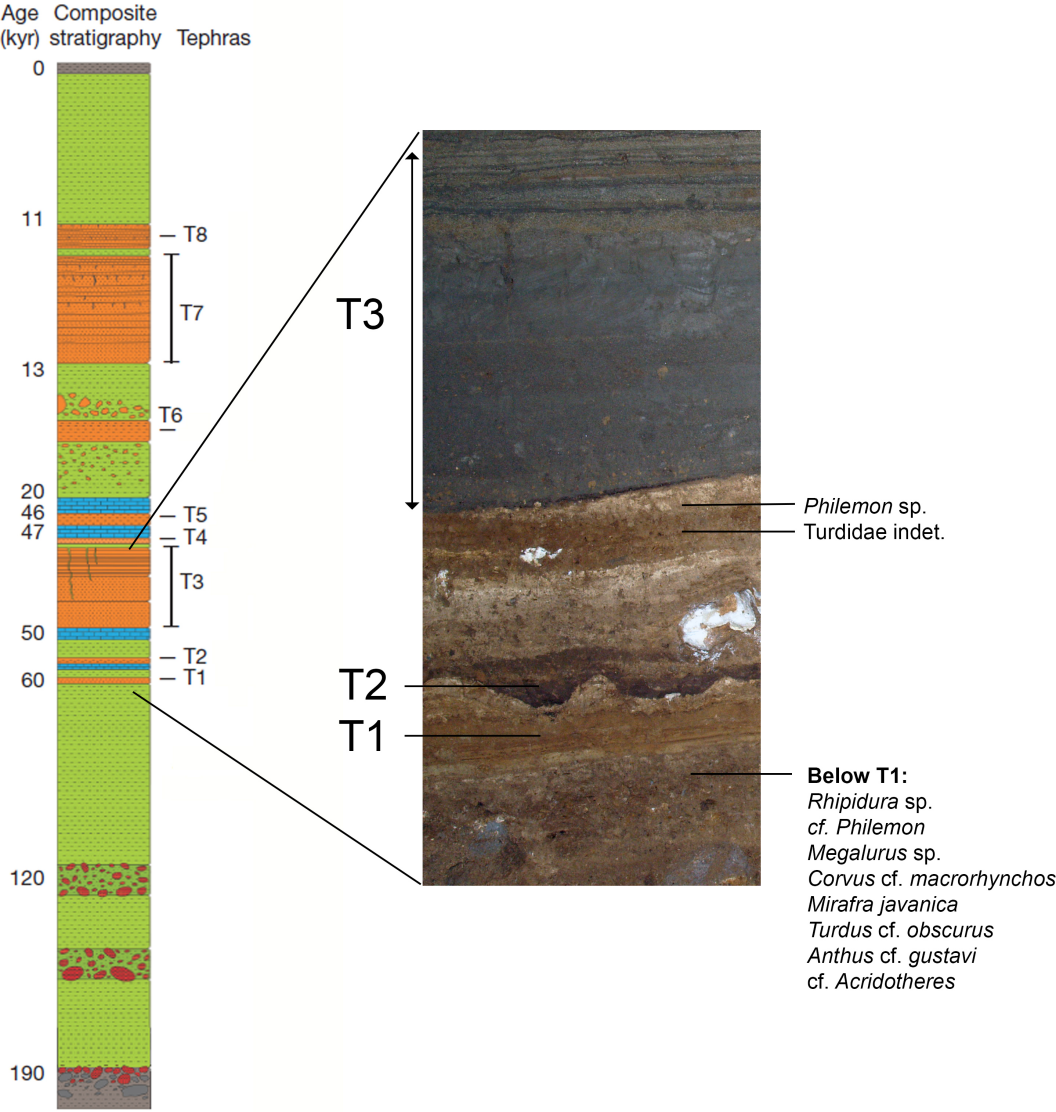
The distribution of passerines within Late Pleistocene deposits of Liang Bua’s Sector XII. Composite stratigraphic section after Sutikna et al. (2016). Sector XII preserves Late Pleistocene sediments between ∼50 and 190 ka, that are capped by volcaniclastic mass flow deposits (Tephra 3, or T3, orange), a key stratigraphic layer in the Liang Bua depositional sequence. The volcanic tephras T1–T3 are separated by calcithic spelothems (blue) and fine-grained clastic sediments (green). T3 varies between ∼0.5 and 0.75 cm thickness throughout the entire sector. T1 directly overlies *Homo floresiensis-* bearing deposits. Approximate dates (in ka) for each unit are given on the left.

## Discussion

The songbirds identified from the Late Pleistocene sediments at Liang Bua (Fig. 5, Table 2) constitute the first fossil passerine sample described from Wallacea. Overall, Liang Bua’s Late Pleistocene songbird assemblage suggests that mixed habitats likely surrounded the cave (Table 2). The fantail cf. *Rhipidura*, friarbird *Philemon* sp., and Eye-browed Thrush *Turdus* cf. *obscurus* are indicative of wooded habitats, whereas the Pechora Pipit *A.* cf. *gustavi* and the Large-billed Crow *Corvus* cf. *macrorhynchos* often frequent open woodlands and forest edges. The Australasian Bushlark *Mirafra javanica* and the grassbird *Megalurus* both occur in grasslands with scattered shrubs, although grassbirds also occur in marsh vegetation. These habitat preferences suggest that a mixture of habitats including open, grassland areas and marshes in addition to tall, closed forests surrounded Liang Bua (Table 2). This agrees with previous work on the Late Pleistocene non-passerine avifauna at Liang Bua that suggested a mixture of nearby habitats, including forests, wetlands and drier grasslands (Meijer et al., 2013). However, the wetland signal observed in the non-passerine avian assemblage is less clear in that of the passerines, which are generally less dependent on aquatic habitats.

Avian bone assemblages in caves may result from fluvial transport, pitfalls, hibernation/aestivation and predation (Andrews, 1990). For the non-passerine assemblage from Liang Bua, raptors were likely responsible for the accumulation of the small bird bone material (Meijer et al., 2013). Remains of barn owls (*Tyto* sp.) have been found in the Late Pleistocene sequence, as well as those of Brahminy kites (*Haliastur* cf. *indus*) and eagles (*Aquila* sp.). With the exception of the Jungle Crow, all passerines identified here fall within the prey size range reported for barn owls (<200 g; Andrews, 1990; Taylor, 1994). Predatory bats may also have contributed to the avian assemblage at Liang Bua and although the presence of predatory bats in the Liang Bua sequence has yet to be confirmed, the extant *Hipposideros diadema* (known from Flores) occasionally eats birds (Pavey & Burwell, 1997).

The living species of *Acridotheres* on Flores, the Javan Myna *A. javanicus,* was first recorded on Flores in 1990 (Coates & Bishop, 1997). Native to Java and Bali, this species has also successfully colonized Peninsular Malaysia, Singapore, Taiwan, Japan, Borneo, Sulawesi and the Lesser Sundas (Tasirin & Fitzsimons, 2014) but is thought to be only recently introduced to Flores (Coates & Bishop, 1997; Verhoeye & Holmes, 1998). The only other species of *Acridotheres* in Wallacea is the Pale-bellied Myna *A. cinereus,* a species endemic to south Sulawesi. Our results suggest that a species of *Acridotheres* was also present at Liang Bua during the Late Pleistocene (LB-Av-930) but it is not yet possible to determine whether this was *A. javanicus*, *A. cinereus*, or possibly an extinct species.

Until this study, the grassbird *Megalurus* has never been recorded on Flores, but *M. timoriensis* is known from the nearby islands of Sumba and Timor (Coates & Bishop, 1997), which are located southwest and east of Flores, respectively. In that light, the absence of *Megalurus* on Flores today is peculiar, and an extinct population would fill the gap in the current distribution of *Megalurus* across the Lesser Sundas. Whether the two specimens referred to *Megalurus* sp. in this study represent different species is unclear: both differ in size, with the larger one being similar in size to *M. timoriensis*. Size varies among *M. timoriensis* subspecies, and males are also larger than females (Madge, 2017a). The two Liang Bua specimens may therefore represent intraspecific rather than interspecific variation. Despite their terrestrial lifestyle, modern *Megalurus* spp. inhabit islands separated by permanent water barriers, indicating that over-water dispersal has occurred. Given that neither Sumba nor Timor were ever connected to Flores by land bridges during the Quaternary, the same must have been true for any past population on Flores. However, extant *M. timoriensis* have never been observed flying across water (Mayr & Diamond, 2001), suggesting that over-water dispersal may be infrequent and possibly limited to immature birds in search of territory (Mayr & Diamond, 2001).

Another species of special interest at Liang Bua, the largest in the passerine assemblage, is the Large-billed Crow *C.* cf. *macrorhynchos*. Crows are highly adaptable and often successfully co-exist with human populations. The occurrence of *C.* cf. *macrorhynchos*—an omnivore known to feed with vultures on carcasses (Madge, 2017b)—in the *H. floresiensis*-bearing layers (Fig. 5) suggests that it formed part of a scavenging guild and fed alongside two large-bodied scavenging birds, the vulture *Trigonoceps* sp. and the extinct giant stork *Leptoptilos robustus* (Meijer & Due, 2010; Meijer et al., 2013). The remains of these birds are closely associated with those of *H. floresiensis*, komodo dragon, and pygmy *Stegodon*, and were likely attracted to the cave by the presence of pygmy *Stegodon* carcasses. A dependence upon megafauna, specifically *S. f. insularis,* is thought to have led to the extinction of both *Trigonoceps* sp. and *L. robustus* on Flores, as a population decline and ultimate disappearance of *S. f. insularis* as a source of carrion would have resulted in a significantly reduced food base for these large carnivorous birds (Meijer et al., 2013). As a smaller, omnivorous bird, the extinction of *S. f. insularis* megafauna would have had a less significant impact on its survival.

The songbirds identified from the Late Pleistocene sediments at Liang Bua constitute the first fossil passerine sample from Wallacea and add significantly to the avian fossil record in Southeast Asia. The remains of these birds increase our knowledge of the palaeoecology of Flores and *H. floresiensis* as well as stimulate new questions about past avian dispersals to Wallacean islands. Further research on the avian skeletal elements preserved at Liang Bua and elsewhere in the greater region is warranted.

##  Supplemental Information

10.7717/peerj.3676/supp-1 Appendix S1 List of extant species and specimens used in comparisons Abbreviations: SMF, Senckenberg Research Institute, Frankfurt, Germany; BM, Bergen University Museum, Bergen, Norway; NMNH, the Smithsonian Institution’s National Museum of Natural History, Washington DC, USA.

10.7717/peerj.3676/supp-2 Supplemental Information 2 A compilation of songs of songbirds identified from Liang Bua’s Pleistocene sediments Species in audio: 0.00–0.14s, *Philemon buceroides*; 0.14–0.26s,* Rhipidura rufifrons*; 0.26–0.42s, *Corvus macrorhynchos*; 0.42–0.56s,* Anthus gustavi*; 0.56–1.07s,* Mirafra javanica*; 1.07–1.17s,* Megalurus timoriensis alisteri*; 1.17–1.30s, * Acridotheres javanicus;* 1.31-1.43s,* Turdus obscurus.* Files are shared under a Attribution-NonCommercial-ShareAlike 4.0 International (CC BY-NC-SA 4.0) license (XC299196, *Turdus obscurus,* Albert Lastukhin & Vadim Ivushkin, http://www.xeno-canto.org/299196; XC284761, *Corvus macrorhynchos*, Anthony Schubert, http://www.xeno-canto.org/284761; XC296857, *Anthus gustavi menzbieri,* Ukolov Ilya, http://www.xeno-canto.org/296857; XC292556 *Philemon buceroides*, Mike Nelson, www.xeno-canto.org/292556 ), or with permission from the recordists (Andrew Spencer, XC312203 *Megalurus timoriensis alisteri*, http://www.xeno-canto.org/312203; Mike Nelson, XC150649 *Acridotheres javanicus*, http://http://www.xeno-canto.org/150649; Frank Lambert, XC201913 *Rhipidura rufifrons*, www.xeno-canto.org/201913 , and XC68725 *Mirafra javanica,* http://www.xeno-canto.org/68725).

## Additional Information and Declarations

### Field Study Permissions

The following information was supplied relating to field study approvals (i.e., approving body and any reference numbers): Excavations were authorised by Pusat Penelitian Arkeologi Nasional (Jakarta, Indonesia).

### Data Availability

The following information was supplied regarding data availability: The raw data is included in the species account section in the manuscript.
