# Supplementary material for: Late Pleistocene songbirds of Liang Bua (Flores, Indonesia); the first fossil passerine fauna described from Wallacea
Source: PeerJ. 2017 Aug 17;5:e3676. doi: 10.7717/peerj.3676 (PMC5563437; doi:10.7717/peerj.3676)
Supplement: Appendix S1 — Abbreviations: SMF, Senckenberg Research Institute, Frankfurt, Germany; BM, Bergen University Museum, Bergen, Norway; NMNH, the Smithsonian Institution’s National Museum of Natural History, Washington DC, USA. [file peerj-05-3676-s001.docx]

**Appendix A. List of extant species used in comparisons.**

**Picidae:** *Dendrocopus major* SMF 1628; **Alcedinidae:** *Alcedo atthis* SMF 2376; **Pittidae**: *Pitta erythrogaster* NMNH 615028; *P. sordida palawanensis* NMNH 562130; **Meliphagidae:** *Lichmera indistincta* NMNH 620254, NMNH 612736; *Melilestes megarhynchus* *megarhynchus* NMNH 489103; *Meliphaga aruensis aruensis* NMNH 489150; *Melitograis. gilolensis* NMNH 557482, NMNH 557475; *Myzomela obscura* NMNH 557469; *Philemon corniculatus* SMF 7245; *P. buceroides* NMNH 347688; **Campephagidae:** *Coracina papuensis* NMNH 557304; *C. pectoralis* SMF 18034; *C. tenuirostris* NMNH 572410; *C. novaehollandiae* NMNH 620247; *Lalage leucopyga montrosieri* NMNH 561831; *Pericocrotus miniatus* NMNH 20317; **Pachycephalidae:** *Pachycephala pectoralis* SMF 4268, NMNH 612732, NMNH 557424, NMNH 612733; *P. nudigula* SMF 2841; *P. cinerea* NMNH 613717; *P. raveni* NMNH 226030; **Oriolidae:** *Oriolus chinensis* SMF 13114, NMNH 635310, NMNH 319492, NMNH 613097, NMNH 635311, NMNH 613095, NMNH 613096, NMNH 613092, SMF 13111, SMF 3005; *Oriolus oriolus* SMF 3813; *O. phaeochromus* NMNH 560850; *O. sagittatus* NMNH 347675; *O. szalayi* NMNH 489064; *O. xanthornus* SMF 169; *O. xanthonotus* *persuasus* NMNH 562061; *Sphecotheres vieilloti* SMF 7316; *S. viridis* NMNH 620242, NMNH 632140; **Artamidae**: *Artamus leucorhynchus* NMNH 20321, NMNH 226022; **Vangidae***: Hemipus picatus* NMNH 290965; **Dicruridae**: *Dicrurus hottentottus* NMNH 561978, NMNH 558326; **Rhipiduridae:** *Rhipidura albicollis* SMF 417, NMNH 620568, 559882; *R. cyaniceps* NMNH 613728; *R. javanica* NMNH 559915; *R. rufifrons* NMNH 556243, 556244; *R. teysmanni* NMNH 226220; **Lanidae:** *Lanius collurio* SMF 5278; **Corvidae:** *Cissa chinensis* SMF 662; *Corvus corone corone* SMF 11355; *C. corax* SMF 7716, SMF 554; *C. enca* NMNH 225830, NMNH 562055; *C. macrorhynchos* NMNH 635306, NMNH 635180, NMNH 612091, NMNH 290457; *C. orru* NMNH 559044, NMNH 558338; *C. typicus* NMNH 226205; *C. validus* NMNH 557300, NMNH 557301; *Dendrocitta vagabundus* SMF 5025; **Monarchidae:** *Monarcha trivirgata* NMNH 557391, NMNH 557398; *Myiagra galeata* NMNH 557413; *Tersiphone atrocaudata* NMNH 560704; *T. viridis* SMF 9120; **Dicaeidae** *Dicaeum aeruginosum affine* NMNH 562135; *D. hypoleucum* NMNH 613193; *D. sanquinolentum* NMNH 20325; **Nectarinidae:** *Anthreptes malacensis* NMNH 488354; *A. malacensis citrinus* NMNH 225837; *Nectarinia jugularis* NMNH 557452, NMNH 557447; *N. venusta* SMF 3688; **Prunellidae:** *Prunella rubeculoides beicki* NMNH 319261, *P. modularis* NMNH 561867; **Estrildidae:** *Amandava amandava* SMF 7550, NMNH 556150, NMNH 556153, NMNH 556155; *Lonchura molucca* NMNH 557486, *L. punctulata cabanisi* NMNH 560917; *L. striata* NMNH 319396; **Passeridae:** *Passer montanus* BM 3778, SMF 13450; **Motacillidae:** *Anthus gustavi gustavi* NMNH 613107, NMNH 613108, NMNH 613109; *A. spinoletta* BM 2173; *A. pratensis* BM 423, BM 7458*; Motacilla alba* BM 7448, B 3187*; M. cinerea* NMNH 292334, NMNH 292271; *M. flava* NMNH 604694, NMNH 611840 ; *M. flava taivana* NMNH 562158; *M. flava tschutschensis*, NMNH 498814, NMNH 498815; **Stenostiridae:** *Culicicapa ceylonensis* NMNH 292965; **Alaudidae:** *Alauda arvensis* BM 7433, BM 7250, SMF 5572; *Mirafra javanica* NMNH 612719, NMNH 612718, NMNH 492470; **Cisticolidae:** *Cisticola exilis grayi* NMNH 226028; *C. juncidis* NMNH 603270, NMNH 604561; **Locustellidae:** *Bradypterus luteoventris* NMNH 318312, NMNH 318311, NMNH 318313; *Cincloramphus cruralis* NMNH 612623; *C. mathewsi* NMNH 561477; *Locustella fasciolata* NMNH 558356; *L. ochotensis* NMNH 560898; *Megalurus palustris forbesi* NMNH 560895, NMNH 613172, NMNH 613171, NMNH 613173; *M. timoriensis* NMNH 488953, NMNH 561990; **Acrocephalidae:** *Acrocephalus orientalis* NMNH 560914; **Pnoepygidae:** *Pnoepyga pusilla* NMNH 620586; **Phylloscopidae:** *Phylloscopus collybita* SMF 5834; *Seicercus burkii* NMNH 620582, NMNH 344894; **Scotocercidae:** *Tesia cyaniventer* NMNH 620583; *T. olivea* NMNH 633716; **Zosteropidae:** *Zosterops chloris intermedius* NMNH 226032; *Z. palpebrosus buxtoni* NMNH 20324; *Z. p. auriventer* NMNH 343385; *Z. montanus* NMNH 648467; *Z. xanthochroa* NMNH 561750; **Sturnidae:** *Acridotheres cristatellus cristatellus* NMNH 122005, NMNH 560912; *A. javanicus* SMF 7784; *A. gingianus* SMF 6664; *A. tristis* SMF 7308; *A. tristis tristis* NMNH 343355; *Ampeliceps coronatus* NMNH 490681; *Aplonis minor* NMNH 488932, NMNH 488934; *Basilornis celebensis* NMNH 226203; *Gracula religiosa* SMF 8638, NMNH 432708; *Leucopsar rothschildi* NMNH 613759; *Mino dumontii*, SMF 2313, NMNH 318504; *Rhabdornis mysticalis* NMNH 607541; *R. inornatus* NMNH 613192, NMNH 607542; *Streptocitta albicollis torquata* NMNH 226204; *Sturnus vulgaris* BM 3023, BM 7175; SMF 5508; **Muscicapidae:** *Brachypteryx montana* NMNH 613709, NMNH 613711; *B. leucophrys* 620543; *Monticola solitaria* NMNH 560700; *Muscicapa thalassina* NMNH 292097; *Niltava rubeculoides* NMNH 561501; *Saxicola caprata* NMNH 226218; **Turdidae:** *Turdus chrysolaus* NMNH 635145, NMNH 635147; *Turdus merula* SMF 5561, SMF 551; *T. merula mandarinus* NMNH 291667, NMNH 292782; *T. naumanni* NMNH 319025; *T. obscurus* NMNH 611771, NMNH 319606, NMNH 344004; *T. ruficollis* NMNH 292784, *T. poliocephalus* NMNH 559860; *Zoothera citrina rubecula* SMF 8767; *Z. dauma* SMF 6540, NMNH 635334, NMNH 635164, NMNH 613714; *Z. interpres* SMF 560; *Z. mollissima* NMNH 319031, NMNH 319296; *Z. gurneyi* NMNH 491223, SMF 9117; *Z. naevia* NMNH 556677, NMNH 556678;
